# Maple Syrup Urine Disease: An Uncommon Cause of Neonatal Febrile Seizures

**DOI:** 10.7759/cureus.40826

**Published:** 2023-06-22

**Authors:** Harshyenee K K, Pranav Ajmera, Aastha Agarwal, Ajay Dahiya, Vinay Kumar Parripati

**Affiliations:** 1 Radiology, Dr. D. Y. Patil Medical College, Hospital & Research Centre, Pune, IND

**Keywords:** gas chromatography mass spectrometry, branched-chain amino acids, maple syrup urine disease, restricted diffusion, magnetic resonance imaging, neonatal febrile seizures

## Abstract

Maple syrup urine disease (MSUD) is a rare autosomal-recessive disorder. An enzyme complex called branched-chain alpha-keto acid dehydrogenase (BCKAD) metabolizes branched-chain amino acids (BCAAs), such as leucine, isoleucine, and valine, in the body. The deficiency of this enzyme causes the accumulation of BCAAs in cerebrospinal fluid, plasma, and urine. This metabolic illness is defined by abnormal levels of BCAAs. The pathognomonic illness marker alloisoleucine is produced in the absence of the BCKAD enzyme, which is part of a metabolic pathway involving three BCAAs and gets accumulated in the body. Classically, affected neonates present with feeding problems, vomiting, lethargy, and irritability, leading to seizures, coma, and death if left untreated. Blood and urine analysis reveals an accumulation of BCAAs in the plasma and urine. Here, we report the case of a neonate on day 10 of life with febrile seizures and non-acceptance of feeds, who was diagnosed with the classical form of MSUD. This is a classic case of MSUD which was evaluated exhaustively and revealed all classic features clinically and on investigations.

## Introduction

Maple syrup urine disease (MSUD) is a rare autosomal-recessive disorder. An enzyme complex called branched-chain alpha-keto acid dehydrogenase (BCKAD) metabolizes branched-chain amino acids (BCAAs), namely, leucine, isoleucine, and valine, in the body. The enzyme deficiency causes the accumulation of BCAAs in cerebrospinal fluid, plasma, and urine. This report describes the detailed blood investigation and imaging evaluation of a neonate, which revealed a pattern typical of MSUD.

Branched-chain ketoaciduria, often known as MSUD, is a metabolic illness defined by abnormal levels of BCAAs. The pathognomonic illness marker alloisoleucine is produced in the absence of the enzyme BCKAD, which is part of a metabolic pathway involving three BCAAs and accumulates in the body [1].

Classically, affected neonates present with feeding problems, vomiting, lethargy, and irritability, leading to seizures, coma, and death if left untreated [2]. Blood and urine analysis reveals an accumulation of BCAAs in the plasma and urine of such patients.

Despite extensive research on PubMed and EBSCOhost, the overall documented number of classical MSUD cases available in the literature is fewer than 50. We report the case of a neonate on day 10 of life with febrile seizures and non-acceptance of feeds, who was diagnosed with the classical form of MSUD.

A neonate with an uncomplicated birth history developed non-acceptance of feeds and suffered from an episode of febrile seizures. Magnetic resonance imaging (MRI) of the brain revealed classical features of MSUD. The diagnosis was confirmed by mass spectroscopy and gas chromatography. The neonate improved on a special diet and had no further seizures for a week.

This classic case of MSUD was evaluated exhaustively and revealed all classic features both clinically and during the investigation. This systematically detailed presentation will provide an example for clinicians and radiologists to review when faced with a diagnostic dilemma and thus ensure this uncommon yet partially treatable disorder is managed timely.

## Case presentation

A female neonate was born in our hospital with an uncomplicated birth history. She was delivered by normal vaginal delivery at term with a 2.5 kg birth weight. At birth, she cried and responded well to external stimuli. The neonate developed non-acceptance of feeds by day three of life and was subsequently kept under continuous observation. On day 10, the neonate suffered from an episode of febrile seizures and was immediately shifted to the neonatal intensive care unit (NICU). In the NICU, she was initiated on intravenous (IV) feeding with dextrose-10 and normal saline (NS at 0.45 bolus), IV calcium gluconate at 2 mL/kg TDS, IV dobutamine at 10 µg/kg/minute, IV cefixime, IV phenobarbital, and IV levetiracetam. A general clinical examination in the NICU revealed that the heart rate, blood pressure, oxygen saturation, and blood sugar levels were within the normal range. Routine hematological investigations were within the normal range for age. On neurological assessment, the neonate revealed hypotonia and poor neonatal reflexes.

**Figure 1:**
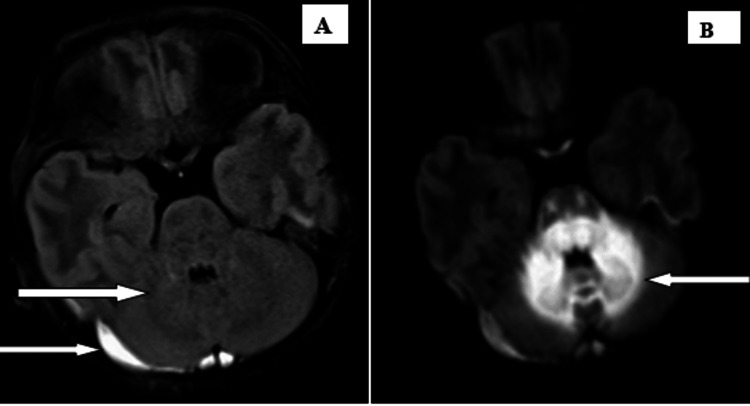
Subtle FLAIR hyperintense are signals noted in the optic chiasma, pons, vermis, and cerebellum. Mild subdural collection is noted in the bilateral cerebellar hemisphere (A). Diffusion restriction is noted in the optic chiasma, pons, vermis, and cerebellum (B). FLAIR: fluid-attenuated inversion recovery

**Figure 2:**
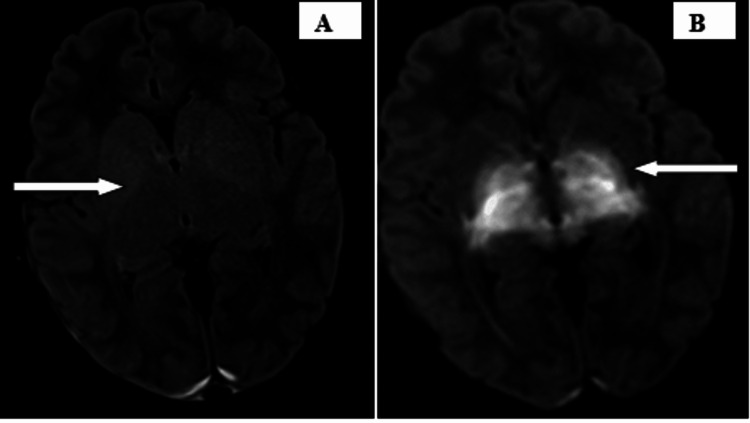
Subtle FLAIR hyperintense signals are noted in the bilateral globus pallidum, thalamus, genu, and the posterior limb of the internal capsule (A). Diffusion restriction is noted in the bilateral globus pallidum, thalamus, genu, and posterior limb of the internal capsule (B). FLAIR: fluid-attenuated inversion recovery

**Figure 3:**
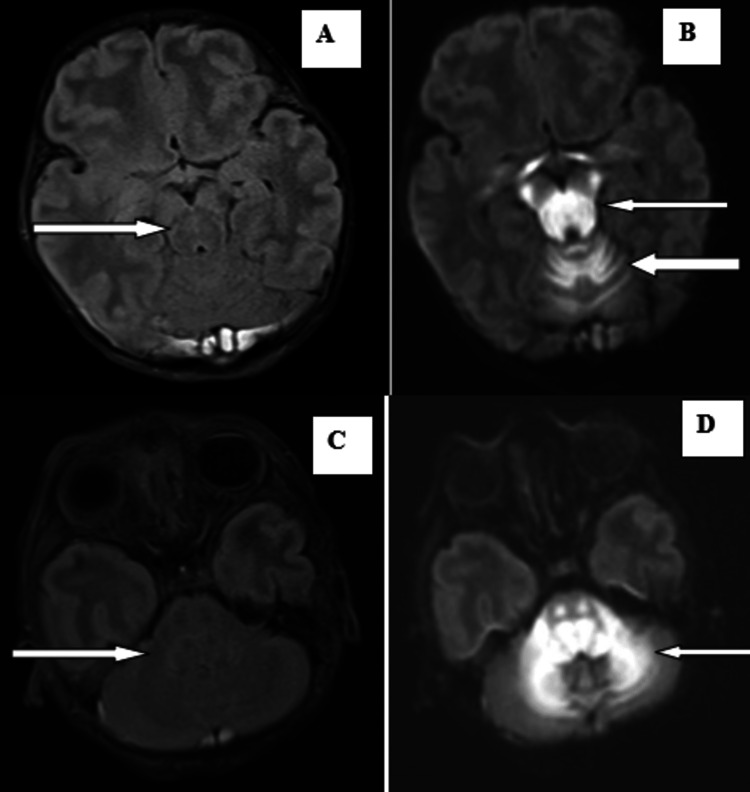
Subtle FLAIR hyperintense signals are noted in the dorsal aspect of the midbrain, pons, medulla, vermis, and cerebellar white matter (A, C). Diffusion restriction is noted in the dorsal aspect of the midbrain, pons, medulla, vermis, and cerebellar white matter (B, D). FLAIR: fluid-attenuated inversion recovery

Subsequently, magnetic resonance imaging (MRI) brain without contrast and magnetic resonance venography were advised on a 3-T scanner. The scan revealed T1 iso to hypointense, subtle T2, or fluid-attenuated inversion recovery (FLAIR) hyperintense areas, showing diffusion restriction with corresponding low apparent diffusion coefficient values, without any blooming on gradient echo sequences involving bilateral peri-rolandic cerebral white matter, also known as the central lobe or paracentral area, which consists of the pre and postcentral gyrus, central sulcus, and the paracentral lobule, bilateral corona radiata, bilateral optic chiasma, and optic radiation (Figure 1); bilateral globus pallidum, thalamus, genu, and posterior limb of the internal capsule; bilateral corticospinal tracts; dorsal aspect of the midbrain and pons; medulla; bilateral cerebral peduncle; vermis; and bilateral cerebellar white matter (Figures 2, 3). A subtle T2 or FLAIR hyperintense signal is noted in the anterior limb of the internal capsule. Mild subdural collection along the bilateral cerebellar hemisphere with a thickness of 6 mm on the right side and 2 mm on the left side MR venography of the brain revealed no additional abnormalities.

**Figure 4:**
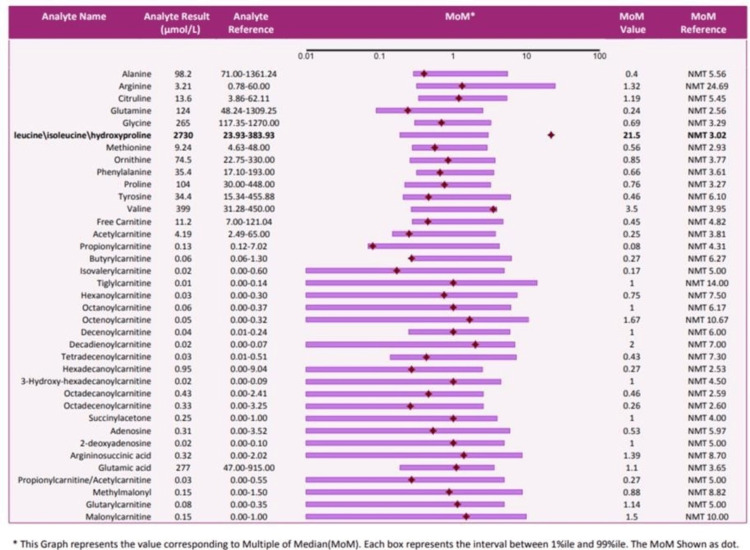
Image showing elevated levels of leucine/isoleucine/hydroxyproline in the blood sample.

Further, the neonate was evaluated with tandem mass spectroscopy and gas chromatography-mass spectrometry, which revealed elevated leucine to phenylalanine levels at a ratio of 77.1 and an increase in the excretion of 2-hydroxy-isovaleric acid and leucine, respectively (Figure 4, Table 1). This confirmed the diagnosis of MSUD.

**Table 1:** Elevated levels of leucine/isoleucine/hydroxyproline in the blood sample.

Analyte name	Analyte result	Analyte reference	Results
Alanine	96.2	71.00–1,361.24	Normal
Arginine	3.21	0.78–60.00	Normal
Citruline	13.6	3.86–62.11	Normal
Glutamine	124	48.24–1,309.25	Normal
Leucine/Isoleucine/Hydroxyproline	2730	23.93–383.93	High
Phenylalanine	35.4	17.10–193.00	Normal

In addition, an EEG was performed, which revealed a comb rhythm. Subsequently, the neonate was started on IV dobutamine at 12 µg/kg/minute, IV Levipil at 10 mg/kg/day, IV Gardenal at 5 mg/kg/day, IV augmentin, and gentamycin.

The neonate was kept hydrated on IV fluids containing dextrose-10 at 180 cc/kg/day. A pediatric dietician was consulted, and a special diet was initiated for the neonate, who showed signs of improvement in the form of a decrease in lethargy and an improvement in feed. The child did not suffer any further episodes of seizures over the next week.

## Discussion

Initially described by Menke in 1954 as a rapidly progressive neurodegenerative disease, the cause of MSUD was described as a deficiency of the BCKAD complex, which functions to break the amino acids leucine, isoleucine, and valine [3]. A deficiency of this enzyme results in the accumulation of these amino acids in the body, which reflects elevated levels in plasma and urine [4]. It is this accumulation that leads to problems with feeding, vomiting, lethargy, and irritability in an affected neonate. Elevation of leucine leads to cytotoxic edema in the brain and affects the myelination of the white matter, leading to seizures and coma [2,4]. The characteristic maple syrup odor of the urine is due to increased isoleucine in the plasma.

MSUD is classified into classic, intermediate, intermittent, E3-deficient, and thiamine-responsive. During the first week of life, individuals with the classic neonatal type exhibit a maple syrup odor in the cerumen and urine. They also develop neurological features such as poor feeding, irritability, apnea, opisthotonos, and “bicycling” movements. Cerebral edema is mostly present, leading to coma and early death. Affected neonates are characterized by having 2% BCKAD enzymatic activity, with such neonates having the worst prognosis.

Infants diagnosed with the intermediate form of the disease typically present later in the first years of their lives with complaints of feeding problems and intellectual disability. Infants diagnosed with intermittent forms show normal growth and neurological development without any symptom onset, even if kept on an unrestricted diet.

However, during catabolic situations, these patients also exhibit the clinical and biochemical characteristics of the classic form, and, in such a situation, they should be treated similarly to those who have the classic type. With treatment, these infants have a better prognosis than individuals with the classic form. The thiamine-responsive form has the best prognosis compared to other affected individuals with classic and intermittent forms of the disease [4,5].

Imaging plays a crucial role in clinching the diagnosis, as MSUD patients have a characteristic pattern of brain edema, particularly during the MSUD crisis. Two forms of edema are seen, namely, intramyelinic edema and vasogenic edema, depending on different pathogenetic mechanisms. In the former, the myelin splits apart due to the BCAAs and water molecules accumulate within the split regions. In the latter, there is a disruption of the blood-brain barrier during an active crisis. The first one has been seen to be the more common cause in classical cases.

The characteristic brain regions involved include cerebellar white matter, peduncles, dorsal brainstem, peri-rolandic cerebral white matter, thalami, globus palladi, and the posterior limb of the internal capsule. These regions appear hypointense on T1-weighted imaging and hyperintense on T2weighted imaging, in accordance with the pathologic edema. Regions with diffusion restriction are particularly prominent in the posterior limb of the internal capsule and corticospinal tract.

MR spectroscopy shows peaks at 0.9-1 ppm during crises, in accordance with the deposition of the BCAAs [6,7].

Leucine, isoleucine, and valine, together with the leucine metabolite alloisoleucine, should be measured as part of the diagnostic procedures for MSUD. Modern newborn screening tests such as tandem mass spectrometry use a single blood sample to check for more than 40 different diseases, plasma amino acid analysis to check for high levels of BCAA and alloisoleucine, and gas chromatography-mass spectrometry to check urine for ketoacids.

Tandem mass spectrometry testing for MSUD assesses both the content of leucine-isoleucine and the ratio of leucine-isoleucine to other amino acids, including alanine, glutamate, glutamine, tryptophan, methionine, histidine, phenylalanine, and tyrosine. Positive cases from gas chromatography-mass spectrometry may require further testing using liquid chromatography-mass spectrometry [4,8].

Management and treatment

Early treatment is preventative against the development of neurological side effects, and such patients usually perform well. Most patients survive and do not develop neurological effects if treated within a few days. However, once neurological symptoms appear, the damage is usually permanent and irreversible. It is important to diagnose the disease as soon as possible and initiate treatment.

Dietary management should be a balance between providing normal growth and development to the patient by giving enough protein and BCAAs and trying to ensure that the patient’s condition and BCAA levels are within normal limits.

Artificially prepared (synthetic) formulas contain all nutrients for proper growth and development but lack BCAAs [9]. A chorionic villus biopsy or amniocentesis may be done in patients with a family history of MSUD to identify the disorder before birth and plan early interventions as needed. Transplantation of the liver from a deceased donor is the most common and frequent approach, but when there is a limitation of a deceased liver donor, a living-related donor liver can be used. An important complication that can occur is post-transplant acute metabolic intoxication because the normal levels of enzyme activity cannot be achieved from deceased unrelated or living-related donor livers [10].

## Conclusions

Children with MSUD should be monitored for at least the first few years of their lives. Regular monitoring of the levels of omega-3 fatty acids, folate, selenium, magnesium, and calcium should be done at least monthly with a metabolic specialist during infancy. At each visit, their progress should be evaluated based on their achievement of milestones that are appropriate for their age.
